# Assessing the usefulness of electroencephalography in psychiatry: Outcome of referrals at a psychiatric hospital

**DOI:** 10.4102/sajpsychiatry.v22i1.702

**Published:** 2016-08-19

**Authors:** Molokashe Molokomme, Ugasvaree Subramaney

**Affiliations:** 1 Netcare Bell Street Hospital, South Africa; 2 Department of Psychiatry, University of the Witwatersrand, South Africa

## Abstract

This retrospective study was conducted at Sterkfontein psychiatric hospital in Gauteng. The objectives included investigating reasons for referral to conduct an electroencephalography (EEG) and to determine whether EEG findings have impact on clinical management. Source data included EEG reports over an 18-month period and clinical records. The total sample was 85 adult inpatients (53 males; 32 females). Seizure disorder exclusion was the main reason for EEG referral (69.0%). Seventy-four (87.0%) records were normal, 7 (8.2%) were abnormal, 2 (2.4%) were inconclusive and 2 (2.4%) EEG reports were unavailable. There was no statistically significant correlation between abnormal EEG results and demographic variables, symptoms, admission diagnosis and medications. EEG recording demonstrated a low yield of abnormal results. In this study, EEG results did not appear to influence the treating psychiatrists regarding management, but this could be as a result of the small sample size. As interactions between psychiatric conditions and epilepsy are important and well established, negative EEGs are indeed useful and it is recommended that clinicians should carefully consider which patients should be referred for EEGs.

The electroencephalography (EEG) has become established as one of the principal investigative tools of cerebral function based on the work by Hans Berger in the 1930s.^1^ It is a non-invasive, low cost, neurodiagnostic technique widely available in general and psychiatric hospitals in South Africa.^2^ Its uses include evaluation of possible organic aetiologies, including the exclusion of seizure disorders and encephalopathic conditions. Earlier studies demonstrated an association between the presence of an organic factor in the history, mental status examination or physical examination and the yield of abnormal EEGs.^3^

Distinguishing between a primary psychiatric disorder and psychiatric manifestations of an underlying medical condition is crucial. This determines which course of management the psychiatrist should follow and, most importantly, determines the prognosis.

EEG does have its limitations. A normal recording does not necessarily exclude pathology. Serial EEGs may have to be done to increase the possibility of a positive finding, which increases the cost of a patient’s workup.^3^

There is limited South African literature on the outcomes of EEG referrals in psychiatry. South Africa’s health service is under-resourced. Assessing the usefulness of EEG in our own setting is necessary.

This study examined reasons for referral to an EEG, outcomes of the referral process in terms of reported abnormalities, correlations between psychiatric signs and symptoms and a positive EEG, and the impact of findings on clinical management.

This was a retrospective record review of patients who were referred for EEG over an 18 month-period at Sterkfontein psychiatric hospital, Gauteng, South Africa. The study was approved by the hospital’s chief executive officer and ethical clearance was obtained from the Human Research and Ethics Committee of the Witwatersrand University (HREC number: M090843). Informed consent was obtained from those who were still inpatients.

Source data included both EEG and clinical records and patients’ demographics, symptoms, admission diagnosis and medications prior to and after EEG. EEG findings and any change in diagnosis after EEG were noted. Data were analysed using Statistica version 9.0. Chi square test and Fischer’s exact test were performed to assess the correlation between abnormal results and the variables.

All patients, except one who had a sleep-deprived EEG, underwent a routine non-sleep-deprived EEG. Photic stimulation and hyperventilation were used during the EEG recordings. All EEGs were performed by one technician with the same EEG machine (Neurofax EEG 1000/9000 Version 05–11) and reported by the same neurologist.

Of the sample of 85 adult inpatients (53 males; 32 females), 74 (87.0%) records were normal, 7 (8.2%) were abnormal, 2 (2.4%) were inconclusive and 2 (2.4%) EEG reports were unavailable. The mean age of the sample was 33.6 (SD 11.5). Figure 1 illustrates the reason for referral to an EEG in percentages.

**FIGURE 1:**
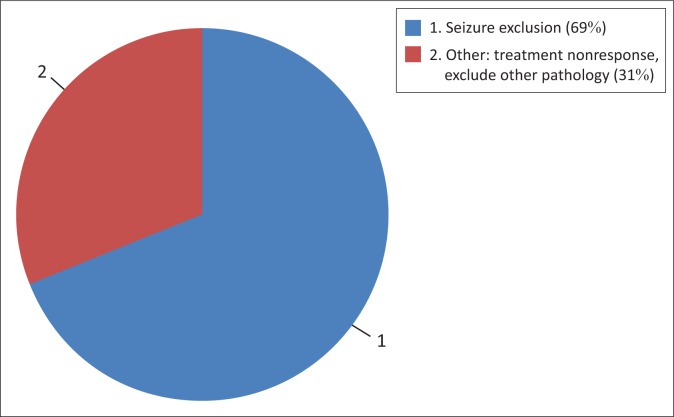
Reason for referral to electroencephalography.

Auditory hallucinations, aggression and visual hallucinations were the most common documented symptoms (Table 1). A history of seizures was reported in 28.2% of the sample population prior to the EEG. EEG abnormalities in the 8.2% (*n* = 7) of the sample population were documented as slowing and dysrhythmia. There was no statistically significant correlation between abnormal EEG results and demographic variables, symptoms (Table 1), admission diagnosis and medications (*p* > 0.05).

**TABLE 1:** Electroencephalography results matched with symptoms.

Symptom	Normal EEG *n*	%	Abnormal EEG *n*	%	*p*
Disorientation	12	14.8	1	1.2	1
AH	45	56.2	4	5	1
VH	35	43.7	4	5	0.7
TH	8	10.3	1	1.3	1
OH	8	10.3	1	1.3	1
Seizure	22	27.2	2	2.5	1
Dysmegalopsia	8	10.3	2	2.56	0.17
Aggression	44	54.3	2	2.5	0.2
Jamais vu/déjà vu	2	2.6	0		1

The finding of no correlation between any of the symptoms and abnormal EEG results is contrary to Szabo’s study of adolescents whereby aggression and hallucinations predicted positive EEG.^4^ In his sample of 36 patients, 44.0% of the patients received a definite diagnosis of complex partial seizures, based on both clinical features and EEG findings. In the remainder, 34.0% had non-specific abnormal EEGs and 22.0% were normal. The clinical features predictive of an EEG abnormality, but not a change in diagnosis or management, included aggression, hallucinations and a premorbid insult.^4^

Of note was that more than 60.0% of the sample already had a suspected or confirmed primary psychiatric disorder or a substance-related disorder without any clear, convincing organic factors on mental status and neurological examinations.

In the present study, only one patient’s diagnosis changed as a result of an abnormal EEG recording (from schizophrenia to temporal lobe epilepsy). One other patient with abnormal results had reported a history of seizures prior to the EEG and was commenced on an antiepileptic drug based on a witnessed seizure a few days after the EEG (EEG results were not available at the time). Warner et al.^5^ who reviewed 190 EEG recordings and charts of psychiatric inpatients also assessed usefulness of screening EEGs, which was defined as EEG results leading to a change in diagnosis and treatment. They also found that while 61% had routine ‘screening EEGs’, with 36 (31.0%) of these screens having an abnormal recording, only 2 (1.7%) led to a change in diagnosis.^5^

Twenty-nine patients (39.1%) with normal results had changes made to their treatment. This was based on clinical picture rather than EEG results.

Other studies,^1,3^ some using larger sample sizes, for example Lam et al.^3^ (*n* = 1147), have yielded similar findings in terms of clinical significance of normal and abnormal results. The presence of an organic factor on history, mental status examination and physical examination were more likely to yield positive results.^3^ The routine use of EEG for psychiatric patients without any presenting organic factors was discouraged by the authors because of the low yield, and negligible clinical significance of normal and abnormal results.^3^

In the O’Sullivan study, the proportion of abnormal EEGs detected from psychiatric sources was less than the combined non-psychiatric-referred patients.^1^ The above studies demonstrate the limitations of EEG use as a routine screening test. The current study is limited by the small sample size and missing data in some records. However, similar to larger studies reviewed, the impact that scalp EEG recording had on patient management was almost negligible.

Because of the retrospective nature of the study, it was difficult to establish specifically how the results were interpreted by the referring clinician. Relying on case records to establish interpretation by assessing whether there was a change in diagnosis or treatment is a limitation.

EEG should ideally be limited to cases where the history, physical examination and mental state examination in combination suggest an ictal phenomenon or encephalopathic condition. Prolonged studies, notwithstanding the cost, with provocation methods may yield more positive results.
